# The Effects of Endocrine Therapies on Lipid Profiles in Chinese Young Women With Early Breast Cancer

**DOI:** 10.3389/fonc.2021.759595

**Published:** 2021-12-01

**Authors:** Yi-Kun Kang, Xue Wang, Nan-Lin Hu, Jian Yue, Yi-Ran Si, Jie Ju, Song-Lin Gao, Peng Yuan

**Affiliations:** Department of VIP Medical Services, National Cancer Center/National Clinical Research Center for Cancer/Cancer Hospital, Chinese Academy of Medical Sciences and Peking Union Medical College, Beijing, China

**Keywords:** endocrine therapy, lipid, breast cancer, premenopausal, ovarian function suppression

## Abstract

This study aimed to evaluate and compare the effects of various endocrine therapies on lipid profiles in young patients with breast cancer. A retrospective, single-center study was performed to investigate the effects of tamoxifen (TAM), tamoxifen plus ovarian function suppression (TAM+OFS), and aromatase inhibitors plus ovarian function suppression (AI+OFS) on lipid profiles during the 60 months of endocrine therapy in hormone receptor-positive patients aged <40 with early breast cancer. The primary endpoint was the cumulative incidence of lipid events, and the secondary endpoints were the changes in lipid profiles. A total of 230 young patients were included with the mean age of 35.7 years old. The patients in TAM group had significantly lower incidence of 5-year lipid events than those in TAM+OFS group (7.4% versus 21.3%; *P*=0.016) and AI+OFS group (7.4% versus 21.6%; *P*=0.009). The incidence of fatty liver was significantly higher in TAM+OFS group than TAM group (52.5%versus 30.9%; *P*=0.043). Lipid events were associated with younger age (odds ratio (OR)=0.865, 95% confidence interval (CI): 0.780-0960; *P*=0.006), higher baseline LDL-C (OR=14.959, 95% CI: 4.379-51.105; *P*<0.001), and use of OFS (OR=3.557, 95% CI: 1.151-10.989; *P*=0.027). Therefore, application of OFS, with younger age and higher baseline LDL-C, may increase the incidence of lipid events in premenopausal breast cancer. More care should be taken for lipid profiles during the endocrine therapy for young breast cancer patients.

## Introduction

Breast cancer is the most common malignant tumor in women as well as one of the three most common cancers worldwide. In all luminal early breast cancer, adjuvant endocrine therapy over the course of 5 to 10 years is now considered as a standard treatment (1). It is widely acknowledged that endocrine sensitivity is directly related to the degree of hormone receptor positivity. For premenopausal patients, the Early Breast Cancer Trialists’ Collaborative Group (EBCTCG) has recommended to use tamoxifen to reduce the recurrence of ER-positive breast cancer (2). For premenopausal patients with high relapse risk (ie, after chemotherapy or age ≤ 35 years old), the addition of ovarian suppression drugs to tamoxifen or aromatase inhibitors (AIs) may enhance the treatment efficacy, according to the SOFT and TEXT trials (3, 4).

Since estrogens have activity in multiple systems, any therapies that result in estrogen deprivation have the various potential toxicity. It has been reported that the side effects of endocrine therapies include impact on the reproductive system (genitourinary irritation, dryness, atrophy, incontinence), the musculoskeletal system (arthralgia, osteopenia, osteoporosis, bone fractures), the cardiovascular system (angina, myocardial and cerebrovascular ischemia), and the central nervous system (fatigue, headache, depression, cognitive dysfunction) (5–8). Recent studies also detect the effect of endocrine therapy on lipid profiles. Significantly higher total cholesterol (TC) and low-density lipoprotein cholesterol (LDL-C) triglyceride (TG) is found in Japanese postmenopausal breast cancer treated with tamoxifen, compared to those received anastrozole and exemestane (9). Another clinical trial based on Chinese postmenopausal breast cancer detects higher incidence of lipid events in the patients undergoing anastrozole and letrozole than those treated with exemestane, indicating that nonsteroidal Ais as adjuvant therapy increased the risk of lipid events (10). Other studies also show negative effects of endocrine treatment on lipid profiles in premenopausal patients with breast cancer (11, 12).

However, only a few studies focus on young breast cancer, and the effects of endocrine therapies on lipid profiles in young patients remains unstudied. This study aimed to evaluate and compare the long-term effects of various endocrine therapies on lipid profiles in young women with early breast cancer.

## Materials and Methods

### Study Design and Patients

This was a retrospective, single-center study performed at the Cancer Hospital of the Chinese Academy of Medical Sciences. We retrospectively included premenopausal women with early-stage breast cancer who started endocrine therapy in our institution from June 1^st^, 2012 to May 31^st^, 2019. The institutional research ethics committee of the Cancer Hospital of the Chinese Academy of Medical Sciences reviewed and approved the study. Written consent was obtained from each participant.

Inclusion criteria: (a) premenopausal women, (b) <40 years old, (c) diagnosed with hormone receptor-positive, stages I to III invasive breast cancer, (d) patients had undergone breast cancer surgery, with or without chemotherapy and radiotherapy. Exclusion criteria: (a) patients diagnosed with low-density lipoprotein cholesterol (LDL-C) level ≥4.14 mmol/L or fatty liver at baseline, (b) those with a previous history of endocrine therapy, (c) those prescribed lipid-lowering medications, (d) those with severe cardiovascular/cerebrovascular diseases or other malignant tumors, (e) lipid profiles data were not available.

Patients were divided into three groups according to the treatment regimens: tamoxifen group (TAM group), tamoxifen plus ovarian function suppression group (TAM+OFS group), and aromatase inhibitors plus ovarian function suppression group (AI+OFS group). OFS included goserelin and leuprorelin. AIs included steroidal AI (exemestane) and nonsteroidal AI (anastrozole/letrozole).

### Data Collection

Demographic characteristics such as age, body mass index (BMI), and medical history was recorded. Physical examination and laboratory tests were collected at baseline and 3, 6, 9, 12, 18, 24, 36, 48, and 60 months after the initiation of endocrine therapy. Serum lipid parameters including total cholesterol (TC), LDL-C, high-density lipoprotein cholesterol (HDL-C), and triglyceride (TG) concentrations were recorded. The results of abdomen ultrasound were also collected for detecting fatty liver.

### Endpoints

The primary endpoint of the study was the cumulative incidence of lipid events during the 60 months of follow-up. The lipid event was defined as an LDL-C level ≥4.14 mmol/L or initiation of lipid-lowering medication, according to the 2007 Chinese guidelines on the prevention and treatment of dyslipidemia in adults (13). The secondary endpoints were the changes in lipid profiles during the 60 months of treatment and incidence of fatty liver, which was diagnosed by abdomen ultrasound.

### Statistical Analysis

The statistical analysis was performed using a commercially available statistical software program SPSS 22.0 (IBM, Chicago, IL, USA). The continuous variables were presented as mean ± standard deviation (SD), and were analyzed using two-way repeated measures analysis of variance (ANOVA) with treatment and time course as factors. The variables including TG, TC, HDL-C, and LDL-C were compared between groups at each time-point as inter-group comparison. These variables were also compared between baseline and each time-point as intra-group comparison. The categorical variables were expressed as number (percentage), and were analyzed using Chi-square test. Univariate logistic regression was used to show the relationship between demographic characteristics and lipid events. Multivariable logistic regression was applied to show the correlation of potential significant characteristics and lipid events. Odds ratio (OR) with 95% confidence interval (CI) was calculated for logistic regression. Subgroup analysis was applied to analyze the effects of different AIs on lipid events and fatty liver incidence. A two-tailed *P* value <0.05 was considered as statistical significance.

## Results

A total of 230 young patients who had undergone breast cancer surgery received postoperative endocrine therapy. The demographic characteristics of the patients were presented in **Table 1**. The mean age ± SD was 35.7 ± 4.3 years old. 81 subjects were treated with tamoxifen, 61 subjects were treated with tamoxifen plus OFS, and 88 subjects were treated with AIs plus OFS. Among patients in AI+OFS group, exemestane was applied in 43 subjects, and anastrozole or letrozole was used in 45 subjects. Similar baseline TC, TG, LDL-C, and HDL-C was detected among the three groups (all *P* value >0.05).

**Table 1:** Basic characteristics of the included patients.

Parameter	Total	TAM group	TAM+OFS group	AI+OFS group
	230	81	61	88
Age/y	35.7 ± 4.3	36.0 ± 4.3	35.5 ± 4.6	35.6 ± 4.0
BMI, kg/m^2^	23.6 ± 3.1	23.4 ± 3.0	23.9 ± 2.9	23.6 ± 3.2
Hypertension (%)	3 (1.3%)	3 (3.7%)	0 (0%)	0 (0%)
Diabetes mellitus (%)	3 (1.3%)	0 (0%)	0 (0%)	3 (3.4%)
Chemotherapy (%)	194 (80.0%)	64 (79.0%)	44 (72.1%)	76 (86.4%)
Radiotherapy (%)	157 (68.3%)	51 (63.0%)	36 (59.0%)	70 (79.6%)
TC, mmol/L	4.22 ± 0.83	4.15 ± 0.79	4.24 ± 0.87	4.26 ± 0.85
TG, mmol/L	1.24 ± 0.64	1.21 ± 0.79	1.23 ± 0.48	1.28 ± 0.61
LDL-C, mmol/L	2.66 ± 0.63	2.56 ± 0.63	2.69 ± 0.67	2.74 ± 0.61
HDL-C, mmol/L	1.40 ± 0.33	1.40 ± 0.32	1.38 ± 0.29	1.42 ± 0.36
Stage (%)				
I	118 (51.3%)	59 (72.8%)	40 (65.6%)	19 (21.6%)
II	74 (32.2%)	20 (24.7%)	16 (26.2%)	38 (43.2%)
III	38 (16.5%)	2 (2.5%)	5 (8.2%)	31 (35.2%)
ER-positive (%)	227 (98.7%)	80 (98.8%)	60 (98.4%)	87 (98.9%)
PR-positive (%)	212 (92.2%)	73 (90.1%)	59 (96.7%)	80 (90.9%)
HER2-positive (%)	50 (21.7%)	18 (22.2%)	13 (21.3%)	19 (21.6%)

All continuous variables were presented as mean ± standard deviation.

TAM, tamoxifen; OFS, ovarian function inhibitor; AI, aromatase inhibitor; BMI, body mass index; TC, total cholesterol; TG, triglyceride; HDL-C, high-density lipoprotein cholesterol; LDL-C, low-density lipoprotein cholesterol; ER, estrogen receptor; PR, progesterone receptor; HER2, human erbB-2 receptor.

**Figure 1:**
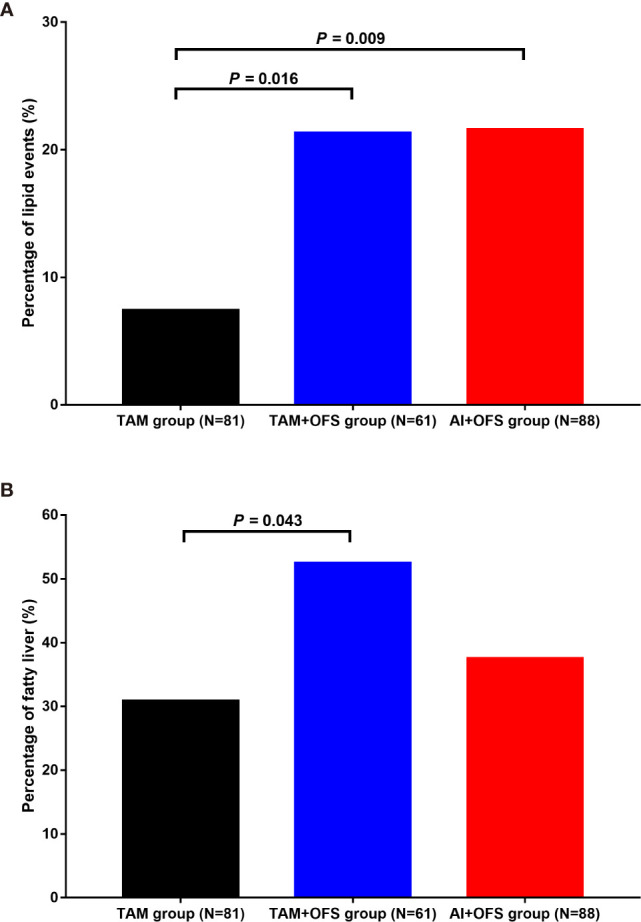
Incidence of lipid events **(A)** and fatty liver **(B)** in different groups. TAM, tamoxifen; OFS, ovarian function inhibitor; AI, aromatase inhibitor.

The patients in TAM group had significantly lower incidence of 5-year lipid events than those in TAM+OFS group (7.4% versus 21.3%; *P*=0.016) and AI+OFS group (7.4% versus 21.6%; *P*=0.009) (**Figure 1**). The 5-year cumulative incidence of fatty liver in the TAM group, TAM+OFS group, and AI+OFS group was 30.9%, 52.5%, and 37.5%, respectively. The incidence of fatty liver was significantly higher in TAM+OFS group than TAM group (*P*=0.043).

Univariate analysis of factors associated with lipid events was shown in **Table 2**. Lipid events were correlated with higher BMI (OR: 1.179, 95% CI: 1.057-1.316; *P*=0.003), baseline TC (OR: 5.263, 95% CI: 2.824-9.807; *P*<0.001), baseline TG (OR: 1.953, 95% CI: 1.203-3.170; *P*=0.007), baseline LDL-C (OR: 12.849, 95% CI: 5.549-29.753; *P*<0.001), and use of OFS (OR: 3.419, 95% CI: 1.364-8.569; *P*=0.009). Lipid events were not correlated with chemotherapy, radiotherapy, baseline LDL-C, tumor subtypes, tumor stage, tamoxifen, and AIs (all *P*>0.05). Potential factors with *P* value lower than 0.10 in univariate logistic regression were further analyzed using multivariate logistic regression (**Table 3**). In multivariate analysis, lipid events were associated with younger age (OR: 0.865, 95% CI: 0.780-0960; *P*=0.006), higher baseline LDL-C (OR: 14.959, 95% CI: 4.379-51.105; *P*<0.001), and use of OFS (OR: 3.557, 95% CI: 1.151-10.989; *P*=0.027).

**Table 2:** Univariate analysis of factors associated with lipid events.

Parameters	OR	95% CI	Regression coefficient B	*P* value
Age	0.928	0.859-1.003	-0.075	0.059
BMI	1.179	1.057-1.316	0.165	0.003
Chemotherapy (reference: no)	1.129	0.462-2.755		0.790
Radiotherapy (reference: no)	0.581	0.284-1.186		0.136
Baseline TC	5.263	2.824-9.807	1.661	0.000
Baseline TG	1.953	1.203-3.170	0.669	0.007
Baseline LDL-C	12.849	5.549-29.753	2.553	0.000
Baseline HDL-C	0.403	0.133-1.220		0.108
ER (reference: negative)	0.389	0.034-4.407		0.446
PR (reference: negative)	1.636	0.360-7.430		0.524
HER2 (reference: negative)	1.870	0.865-4.042		0.111
Stage	1.071	0.675-1.698		0.772
Tamoxifen (reference: negative)	0.561	0.278-1.131		0.106
OFS (reference: negative)	3.419	1.364-8.569	1.229	0.009
AI (reference: negative)	1.783	0.884-3.593		0.106
Exemestane (reference: negative)	1.442	0.626-3.323		0.390
Anastrozole/letrozole (reference: negative)	1.602	0.713-3.600		0.254

OR, odds ratio; CI, confidence interval; BMI, body mass index; TC, total cholesterol; TG, triglyceride; HDL-C, high-density lipoprotein cholesterol; LDL-C, low-density lipoprotein cholesterol; ER, estrogen receptor; PR, progesterone receptor; HER2, human erbB-2 receptor; OFS, ovarian function inhibitor; AI, aromatase inhibitor.

**Table 3:** Multivariate analysis of factors associated with lipid events.

Parameters	OR	95% CI	Regression coefficient B	*P* value
Age	0.865	0.780-0960	-0.145	0.006
BMI	1.081	0.942-1.240		0.268
Baseline TC	0.949	0.446-2.021		0.892
Baseline TG	1.420	0.757-2.663		0.275
Baseline LDL-C	14.959	4.379-51.105	2.705	0.000
OFS (reference: negative)	3.557	1.151-10.989	1.269	0.027

OR, odds ratio; CI, confidence interval; BMI, body mass index; TC, total cholesterol; TG, triglyceride; LDL-C, low-density lipoprotein cholesterol; OFS, ovarian function inhibitor.

The changes of lipid profiles during the treatment were illustrated in **Tables 4**–**7**. TC level was significantly lower in TAM group than AI+OFS group during 6 months to 60 months (all *P*<0.05). Compared to the baseline, TC level in AI+OFS group significantly increased after 18 months (all *P*<0.05). Significant difference of TG level was found between TAM group and TAM+OFS group at 36 months and 60 months (both *P*<0.05). Significant difference of TG level was also detected between AI+OFS group and TAM+OFS group at 9 months and 36 months (both *P*<0.05). Patients in AI+OFS group had significantly lower HDL-C than the other two groups at each time-point except baseline (all *P*<0.05). Compared to the baseline, HDL-C level in TAM group significantly increased after 18 months (all *P*<0.05). LDL-C was significantly higher in AI+OFS group than the other two groups at each time-point except baseline (all *P*<0.05). Compared to the baseline, HDL-C level in AI+OFS group significantly increased after 6 months (all *P*<0.05).

**Table 4:** Comparison of total cholesterol (TC) level at different time-points over the course of treatment.

Group	Baseline	3 months	6 months	9 months	12 months	18 months	24 months	36 months	48 months	60 months
TAM group	4.15 ± 0.79	4.31 ± 0.83	4.22 ± 1.00	4.11 ± 0.76	4.10 ± 0.85	4.22 ± 0.85	4.30 ± 0.76	4.18 ± 0.64	4.36 ± 0.75^#^	4.30 ± 0.75^#^
(n)	(81)	(56)	(51)	(39)	(64)	(52)	(59)	(48)	(38)	(23)
TAM+OFS group	4.25 ± 0.87	4.42 ± 0.91	4.42 ± 0.82	4.30 ± 0.83	4.23 ± 0.75	4.37 ± 0.76	4.37 ± 0.74	4.40 ± 0.68*	4.58 ± 0.99*^#^	4.36 ± 0.89
(n)	(61)	(48)	(42)	(38)	(49)	(42)	(50)	(41)	(39)	(29)
F_inter-group_=7.288, *P*_inter-group_<0.05;F_intra-group_=4.353, *P*_intra-group_<0.001										
TAM group	4.15 ± 0.79	4.31 ± 0.82	4.22 ± 1.00	4.11 ± 0.76	4.10 ± 0.85	4.22 ± 0.85	4.30 ± 0.76	4.18 ± 0.64	4.36 ± 0.75^#^	4.30 ± 0.75^#^
(n)	(81)	(56)	(51)	(39)	(64)	(52)	(59)	(48)	(38)	(23)
AI+OFS group	4.26 ± 0.85	4.47 ± 0.90	4.55 ± 0.84*#	4.48 ± 0.73*	4.48 ± 0.85*	4.50 ± 0.90*^#^	4.60 ± 0.92*^#^	4.70 ± 1.05*^#^	4.61 ± 0.95*^#^	4.87 ± 0.93*^#^
(n)	(88)	(73)	(70)	(58)	(76)	(68)	(67)	(58)	(48)	(37)
F_inter-group_=77.203, *P*_inter-group_<0.001;F_intra-group_=13.443, *P*_intra-group_<0.001										
TAM+OFS group	4.25 ± 0.87	4.42 ± 0.91	4.42 ± 0.82	4.30 ± 0.83	4.23 ± 0.75	4.37 ± 0.76	4.37 ± 0.74	4.40 ± 0.68	4.58 ± 0.99^#^	4.36 ± 0.89
(n)	(61)	(48)	(42)	(38)	(49)	(42)	(50)	(41)	(39)	(29)
AI+OFS group	4.26 ± 0.85	4.47 ± 0.90	4.55 ± 0.84^#^	4.48 ± 0.73	4.48 ± 0.85*	4.50 ± 0.90^#^	4.60 ± 0.92^#^	4.70 ± 1.05*^#^	4.61 ± 0.95^#^	4.87 ± 0.93*^#^
(n)	(88)	(73)	(70)	(58)	(76)	(68)	(67)	(58)	(48)	(37)
F_inter-group_=19.462, *P*_inter-group_<0.001;F_intra-group_=3.696, *P*_intra-group_<0.001										

n represents patient number.

*P value < 0.05, compared to the other group at the same time-point.

^#^P value < 0.05, compared to the baseline at the same group.

**Table 5:** Comparison of triglyceride (TG) level at different time-points over the course of treatment.

Group	Baseline	3 months	6 months	9 months	12 months	18 months	24 months	36 months	48 months	60 months
TAM group	1.21 ± 0.79	1.39 ± 0.88	1.43 ± 1.37	1.26 ± 0.68	1.37 ± 0.97	1.28 ± 0.74	1.23 ± 0.75	1.21 ± 0.55	1.24 ± 0.72	1.23 ± 0.55
(n)	(81)	(56)	(51)	(39)	(64)	(52)	(59)	(48)	(39)	(23)
TAM+OFS group	1.23 ± 0.48	1.74 ± 1.86^#^	1.45 ± 0.61^#^	1.23 ± 0.57	1.41 ± 0.70	1.38 ± 0.64	1.48 ± 1.00^#^	1.64 ± 0.86*^#^	1.41 ± 0.71	1.40 ± 0.58*^#^
(n)	(61)	(48)	(42)	(38)	(49)	(43)	(50)	(41)	(39)	(29)
F_inter-group_=12.858, *P*_inter-group_<0.001;F_intra-group_=2.636, *P*_intra-group_<0.05										
TAM group	1.21 ± 0.79	1.39 ± 0.88	1.43 ± 1.37	1.26 ± 0.68	1.37 ± 0.97	1.28 ± 0.74	1.23 ± 0.75	1.21 ± 0.55	1.24 ± 0.72	1.23 ± 0.55
(n)	(81)	(56)	(51)	(39)	(64)	(52)	(59)	(48)	(39)	(23)
AI+OFS group	1.28 ± 0.61	1.75 ± 2.02^#^	1.53 ± 1.54	1.60 ± 1.53*^#^	1.43 ± 0.90	1.38 ± 1.02	1.46 ± 1.33	1.35 ± 0.81	1.38 ± 0.67	1.28 ± 0.66
(n)	(88)	(73)	(70)	(58)	(76)	(69)	(67)	(58)	(48)	(37)
F_inter-group_=11.201, *P*_inter-group_<0.001;F_intra-group_=2.029, *P*_intra-group_<0.05										
TAM+OFS group	1.23 ± 0.48	1.74 ± 1.86^#^	1.45 ± 0.61^#^	1.23 ± 0.57	1.41 ± 0.70	1.38 ± 0.64	1.48 ± 1.00^#^	1.64 ± 0.86^#^	1.41 ± 0.71	1.40 ± 0.58^#^
(n)	(61)	(48)	(42)	(38)	(49)	(43)	(50)	(41)	(39)	(29)
AI+OFS group	1.28 ± 0.61	1.75 ± 2.02^#^	1.53 ± 1.54	1.60 ± 1.53*^#^	1.43 ± 0.90	1.38 ± 1.02	1.46 ± 1.33	1.35 ± 0.81*	1.38 ± 0.67	1.28 ± 0.66
(n)	(88)	(73)	(70)	(58)	(76)	(69)	(67)	(58)	(48)	(37)
F_inter-group_=0.013, *P*_inter-group_>0.05;F_intra-group_=2.423, *P*_intra-group_<0.05										

n represents patient number.

*P value < 0.05, compared to the other group at the same time-point.

^#^P value < 0.05, compared to the baseline at the same group.

**Table 6:** Comparison of high-density lipoprotein cholesterol (HDL-C) level at different time-points over the course of treatment.

Group	Baseline	3 months	6 months	9 months	12 months	18 months	24 months	36 months	48 months	60 months
TAM group	1.40 ± 0.32	1.49 ± 0.34	1.48 ± 0.36	1.51 ± 0.38	1.53 ± 0.36	1.60 ± 0.40#	1.59 ± 0.35#	1.59 ± 0.43#	1.64 ± 0.42#	1.61 ± 0.34#
(n)	(81)	(56)	(51)	(39)	(64)	(52)	(59)	(48)	(39)	(23)
TAM+OFS group	1.38 ± 0.29	1.46 ± 0.40	1.54 ± 0.34	1.62 ± 0.43#	1.55 ± 0.43	1.64 ± 0.45#	1.58 ± 0.46	1.58 ± 0.49	1.65 ± 0.49#	1.53 ± 0.41
(n)	(61)	(48)	(42)	(38)	(49)	(42)	(50)	(41)	(39)	(29)
F_inter-group_=0.157, *P*_inter-group_>0.05;F_intra-group_=7.392, *P*_intra-group_<0.001										
TAM group	1.40 ± 0.32	1.49 ± 0.34	1.48 ± 0.36	1.51 ± 0.38	1.53 ± 0.36	1.60 ± 0.40#	1.59 ± 0.35#	1.59 ± 0.43#	1.64 ± 0.42#	1.61 ± 0.34#
(n)	(81)	(56)	(51)	(39)	(64)	(52)	(59)	(48)	(39)	(23)
AI+OFS group	1.42 ± 0.36	1.26 ± 0.31*#	1.28 ± 0.30*	1.28 ± 0.32*	1.33 ± 0.37*	1.32 ± 0.35*	1.33 ± 0.35*	1.37 ± 0.38*	1.32 ± 0.34*	1.32 ± 0.30*
(n)	(88)	(73)	(70)	(58)	(76)	(68)	(67)	(58)	(48)	(37)
F_inter-group_=246.318, *P*_inter-group_<0.001;F_intra-group_=4.126, *P*_intra-group_<0.001										
TAM+OFS group	1.38 ± 0.29	1.46 ± 0.40	1.54 ± 0.34	1.62 ± 0.43#	1.55 ± 0.43	1.64 ± 0.45#	1.58 ± 0.46	1.58 ± 0.49	1.65 ± 0.49#	1.53 ± 0.41
(n)	(61)	(48)	(42)	(38)	(49)	(42)	(50)	(41)	(39)	(29)
AI+OFS group	1.42 ± 0.36	1.26 ± 0.31*#	1.28 ± 0.30*	1.28 ± 0.32*	1.33 ± 0.37*	1.32 ± 0.35*	1.33 ± 0.35*	1.37 ± 0.38*	1.32 ± 0.34*	1.32 ± 0.30*
(n)	(88)	(73)	(70)	(58)	(76)	(68)	(67)	(58)	(48)	(37)
F_inter-group_=131.890, *P*_inter-group_<0.001;F_intra-group_=2.647, *P*_intra-group_<0.05										

n represents patient number.

*P value < 0.001, compared to the other group at the same time-point.

^#^P value < 0.001, compared to the baseline at the same group.

**Table 7:** Comparison of low-density lipoprotein cholesterol (LDL-C) level at different time-points over the course of treatment.

Group	Baseline	3 months	6 months	9 months	12 months	18 months	24 months	36 months	48 months	60 months
TAM+OFS group	2.56 ± 0.63	2.60 ± 0.75	2.53 ± 0.75	2.36 ± 0.63^#^	2.39 ± 0.67	2.54 ± 0.72	2.54 ± 0.74	2.42 ± 0.57	2.53 ± 0.67	2.78 ± 0.69^#^
(n)	(81)	(56)	(51)	(39)	(64)	(52)	(59)	(48)	(39)	(23)
TAM group	2.69 ± 0.67	2.66 ± 0.58	2.63 ± 0.73	2.57 ± 0.60*	2.51 ± 0.62	2.63 ± 0.56	2.64 ± 0.67	2.62 ± 0.62*	2.91 ± 0.84*^#^	2.74 ± 0.66
(n)	(61)	(48)	(42)	(38)	(49)	(42)	(50)	(41)	(39)	(29)
F_inter-group_=18.179, *P*_inter-group_<0.001;F_intra-group_=4.540, *P*_intra-group_=<0.001										
TAM group	2.56 ± 0.63	2.60 ± 0.75	2.53 ± 0.75	2.36 ± 0.63^#^	2.39 ± 0.67	2.54 ± 0.72	2.54 ± 0.74	2.42 ± 0.57	2.53 ± 0.67	2.78 ± 0.69^#^
(n)	(81)	(56)	(51)	(39)	(64)	(52)	(59)	(48)	(39)	(23)
AI+OFS group	2.74 ± 0.61	2.90 ± 0.76*	2.96 ± 0.69*^#^	2.88 ± 0.71*	2.95 ± 0.75*^#^	3.00 ± 0.82*^#^	3.06 ± 0.81*^#^	3.21 ± 0.89*#	3.21 ± 0.80*^#^	3.28 ± 0.73*^#^
(n)	(88)	(74)	(70)	(58)	(76)	(68)	(67)	(58)	(48)	(37)
F_inter-group_=288.630, *P*_inter-group_<0.001;F_intra-group_=11.172, *P*_intra-group_<0.001										
TAM+OFS group	2.69 ± 0.67	2.66 ± 0.58	2.63 ± 0.73	2.57 ± 0.60	2.51 ± 0.62	2.63 ± 0.56	2.64 ± 0.67	2.62 ± 0.62	2.91 ± 0.84^#^	2.74 ± 0.66
(n)	(61)	(48)	(42)	(38)	(49)	(42)	(50)	(41)	(39)	(29)
AI+OFS group	2.74 ± 0.61	2.90 ± 0.76*	2.96 ± 0.69*^#^	2.88 ± 0.71*^#^	2.95 ± 0.75*^#^	3.00 ± 0.82*^#^	3.06 ± 0.81*^#^	3.21 ± 0.89*^#^	3.21 ± 0.80*^#^	3.28 ± 0.73*^#^
(n)	(88)	(74)	(70)	(58)	(76)	(68)	(67)	(58)	(48)	(37)
F_inter-group_=105.381, *P*_inter-group_<0.001;F_intra-group_=5.512, *P*_intra-group_<0.001										

n represents patient number.

*P value < 0.05, compared to the other group at the same time-point.

^#^P value < 0.05, compared to the baseline at the same group.

**Figure 2:**
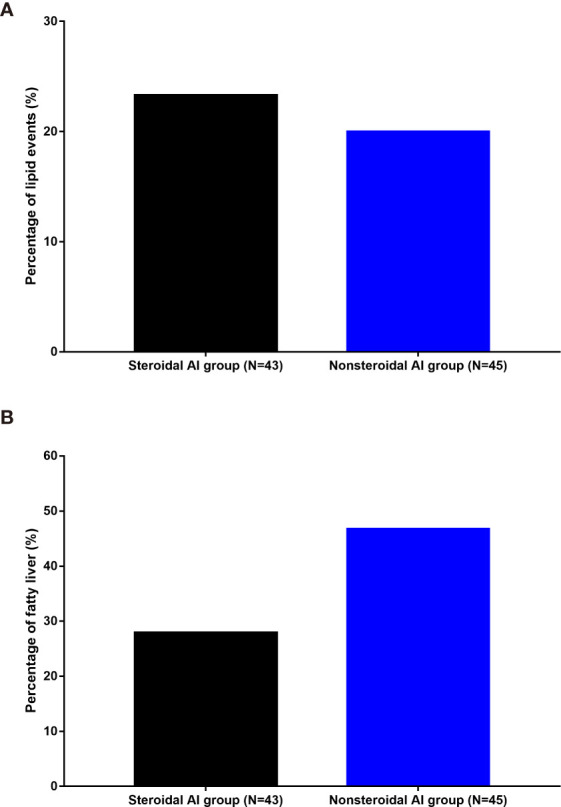
Incidence of lipid events **(A)** and fatty liver **(B)** in different AI groups. AI, aromatase inhibitor.

Subgroup analysis detected no significant difference of lipid events as well as fatty liver incidence between steroidal AI and nonsteroidal AI (both *P*>0.05) (**Figure 2**).

## Discussion

This study shows that lipid disorder is a common side effect of endocrine therapy for young women with breast cancer. Application of OFS, with younger age and higher baseline LDL-C, may further increase the incidence of lipid events. Combined with the previous knowledge on postmenopausal patients with breast cancer, our results demonstrate that endocrine therapy-induced lipid disorders occur not only in postmenopausal subjects, but also in premenopausal women.

Tamoxifen is a selective estrogen receptor modulator widely used in the treatment of breast cancer. In this study, patients treated with TAM alone seems to have a lower risk of lipid events, which is consistent with some previous studies based on postmenopausal women. Tominaga et al. (14) found that the tamoxifen group showed significantly decreased TC and LDL-C levels compared with baseline in Japanese postmenopausal breast cancer. Markopoulos et al. (15) also reported that tamoxifen exerted positive effect on LDL-C levels, however, tamoxifen on the other hand increased TG levels, which is not shown in our study. Other than TG, TC, HDL-C, and LDL-C, another meta-analysis of five studies with 215 participants also found a statistically significant reduction of lipoprotein(a) levels following tamoxifen treatment (16). The beneficial effects of tamoxifen on lipid profiles may possibly result from its agonistic estrogenic activity (17).

Adjuvant therapy with the third-generation AIs, including anastrozole, letrozole, and exemestane, has largely replaced the use of tamoxifen as standard adjuvant endocrine treatment for early breast cancer (18). Although various kinds of AIs have different effects on lipid profiles, current guidelines consider all AIs to have similar cardiovascular and lipid effects (19). In general, endocrine therapy with AIs have a negative impact on serum lipids and may ultimately lead to cardiovascular issues (20, 21). The potential mechanisms of cardiometabolic effects of AIs include changes on body composition, hepatic fat accumulation, glucose metabolism, arterial wall, et al. (11). An open, randomized, multi-center study compared the lipid effects of letrozole, exemestane and anastrozole in healthy postmenopausal women. It showed that exemestane significantly increased the LDL-C:HDL-C ratio by 12 and 24 weeks compared with anastrozole or letrozole (22). In the premenopausal patients, we also find AI plus OFS may increase the risk of lipid events, however, further logistic analysis showed this impact may result from OFS rather than AI. Subgroup analysis also detected no significant difference of lipid events as well as fatty liver incidence between steroidal AI and nonsteroidal AI, which is not consistent with former studies based on postmenopausal breast cancer. A clinical trial performed by Wang et al. showed that a significantly higher cumulative incidence of lipid events occurred in the nonsteroidal AI group than in the steroidal AI group, indicating that steroidal AIs exerted a protective effect against blood lipid events in postmenopausal women receiving an AI as adjuvant therapy for breast cancer (10). The divergence may be due to the different age and menstrual status, and further studies are needed to cover this issue.

The Asian Breast Cancer Cooperative Group (ABCCG) contends that high-risk patients (premenopausal) should receive adjuvant chemotherapy and OFS plus AI or tamoxifen (23). In this study, use of OFS is an important risk factor to lipid events, which is commonly accepted. Lipid metabolism constitutes a key component in energy homeostasis, and estrogens show a powerful control on lipid metabolism by regulating metabolic pathways (24). Therefore, lipid metabolic disorders always occur after menopause due to hormonal changes, such as decreased levels of estrogens and increased levels of circulating androgens (25). Our study further proves that lipid disorders may also occur even in young women when ovarian function is suppressed.

Considering that lipid profiles are predictive factors for coronary heart disease (CAD), more care should be taken for lipid profiles during the endocrine therapy for young patients with breast cancer. Lipid-lowering therapy not only helps patients avoid to suffer from CAD, but also improves the overall survival (OS). A retrospective cohort study examined the impact of statin use on the outcomes of 1523 women diagnosed with operable breast cancer, suggesting that long-term statin use (> 5 years) was associated with improved OS and disease-free survival (DFS) in breast cancer regardless of receptor subtype, even after adjusting for metabolic comorbidities (26).

With broader application of endocrine therapy in young breast cancer, this study adds important information of endocrine therapy-induced lipid disorder during the treatment. Other than endocrine therapy, recent studies also reveal chemotherapy has side effects on lipid profiles in patients with breast cancer (12). Further studies detect chemotherapy had much more effects on lipid profiles in premenopausal women compared to postmenopausal women (27). Therefore, attention should be placed to observe the changes of lipid profiles during various treatment in young breast cancer.

Some limitations should be also noted. First, this is a retrospective study with relatively small sample size. Second, all the included subjects were Chinese and were from a single center, limiting the applicability of the results to patients worldwide. Third, with the various combinations of regimen, we failed to detect the effect of each drug separately. Fourth, we failed to evaluate the cardiometabolic effects of endocrine treatment in this cohort. in addition, we did not study the underlying mechanisms of the discrepancy in lipid disorders between premenopausal and postmenopausal breast cancer.

In conclusion, application of endocrine therapy may increase the incidence of lipid disorders in premenopausal breast cancer. Physicians should make treatment strategies after evaluating the risk of lipid metabolism to avoid severe systemic disorders.

## Data Availability Statement

The original contributions presented in the study are included in the article/supplementary material. Further inquiries can be directed to the corresponding author.

## Ethics Statement

The studies involving human participants were reviewed and approved by Chinese Academy of Medical Sciences. The patients/participants provided their written informed consent to participate in this study. Written informed consent was obtained from the individual(s) for the publication of any potentially identifiable images or data included in this article.

## Author Contributions

Study design, PY. Data collection, YKK. Data analysis, YKK, XW, NLH, JY, YRS, JJ, and SLG. Writing the initial version of the manuscript, YKK. All the authors contributed to revise the draft and gave their final approval for submission of the manuscript for publication.

## Funding

This study was supported by the National Natural Science Foundation of China (81672634, 82172650), Beijing Medical Award Foundation (YXJL-2020-0941-0763).

## Conflict of Interest

The authors declare that the research was conducted in the absence of any commercial or financial relationships that could be construed as a potential conflict of interest.

## Publisher’s Note

All claims expressed in this article are solely those of the authors and do not necessarily represent those of their affiliated organizations, or those of the publisher, the editors and the reviewers. Any product that may be evaluated in this article, or claim that may be made by its manufacturer, is not guaranteed or endorsed by the publisher.
